# Supplementary material for: Variations in the reproductive strategies of three populations of Phrynocephalus helioscopus in China
Source: PeerJ. 2018 Oct 24;6:e5705. doi: 10.7717/peerj.5705 (PMC6203940; doi:10.7717/peerj.5705)
Supplement: Table S1 [file peerj-06-5705-s001.docx]

| Population | EL | EW | EM | SVL | TBW | CS |
| --- | --- | --- | --- | --- | --- | --- |
| BT | 14.30 | 8.69 | 0.54 | 46.70 | 7.26 | 2 |
| BT | 15.94 | 8.71 | 0.60 | 46.70 | 7.26 | 2 |
| BT | 16.42 | 9.02 | 0.60 | 46.15 | 6.73 | 2 |
| BT | 17.55 | 7.83 | 0.59 | 46.15 | 6.73 | 2 |
| BT | 14.76 | 8.17 | 0.48 | 45.51 | 6.88 | 3 |
| BT | 16.00 | 8.26 | 0.52 | 45.51 | 6.88 | 3 |
| BT | 15.08 | 8.31 | 0.56 | 45.51 | 6.88 | 3 |
| BT | 14.18 | 8.19 | 0.51 | 42.83 | 8.32 | 2 |
| BT | 14.26 | 8.58 | 0.59 | 42.83 | 8.32 | 2 |
| BT | 15.60 | 8.31 | 0.58 | 45.24 | 6.22 | 3 |
| BT | 15.36 | 8.27 | 0.58 | 45.24 | 6.22 | 3 |
| BT | 15.74 | 8.42 | 0.59 | 45.24 | 6.22 | 3 |
| BT | 15.54 | 8.77 | 0.46 | 45.51 | 5.89 | 4 |
| BT | 17.50 | 7.72 | 0.57 | 45.51 | 5.89 | 4 |
| BT | 14.47 | 8.35 | 0.48 | 45.51 | 5.89 | 4 |
| BT | 15.69 | 7.72 | 0.46 | 45.51 | 5.89 | 4 |
| BT | 16.42 | 8.95 | 0.57 | 47.75 | 6.93 | 2 |
| BT | 18.51 | 9.03 | 0.76 | 47.75 | 6.93 | 2 |
| BT | 13.57 | 7.94 | 0.47 | 43.94 | 6.80 | 3 |
| BT | 12.52 | 8.23 | 0.47 | 43.94 | 6.80 | 3 |
| BT | 14.66 | 7.98 | 0.49 | 43.94 | 6.80 | 3 |
| BT | 15.48 | 7.25 | 0.52 | 46.34 | 7.34 | 2 |
| BT | 16.07 | 7.42 | 0.53 | 46.34 | 7.34 | 2 |
| BT | 14.36 | 8.06 | 0.53 | 46.72 | 6.25 | 3 |
| BT | 13.89 | 8.52 | 0.50 | 46.72 | 6.25 | 3 |
| BT | 15.56 | 7.72 | 0.51 | 46.72 | 6.25 | 3 |
| BT | 13.32 | 7.80 | 0.24 | 48.00 | 7.26 | 4 |
| BT | 12.47 | 8.64 | 0.39 | 48.00 | 7.26 | 4 |
| BT | 14.17 | 7.77 | 0.37 | 48.00 | 7.26 | 4 |
| BT | 13.16 | 7.65 | 0.30 | 48.00 | 7.26 | 4 |
| BT | 14.08 | 7.34 | 0.42 | 46.80 | 6.38 | 2 |
| BT | 15.86 | 7.70 | 0.51 | 46.80 | 6.38 | 2 |
| BT | 13.20 | 8.31 | 0.45 | 46.99 | 7.78 | 3 |
| BT | 12.77 | 8.51 | 0.47 | 46.99 | 7.78 | 3 |
| BT | 15.00 | 8.47 | 0.56 | 46.99 | 7.78 | 3 |
| FY | 14.96 | 8.41 | 0.63 | 48.42 | 8.15 | 4 |
| FY | 14.21 | 8.54 | 0.62 | 48.42 | 8.15 | 4 |
| FY | 14.53 | 8.34 | 0.51 | 48.42 | 8.15 | 4 |
| FY | 11.49 | 6.90 | 0.27 | 48.42 | 8.15 | 4 |
| FY | 14.82 | 9.39 | 0.70 | 55.26 | 8.23 | 4 |
| FY | 15.30 | 8.88 | 0.69 | 55.26 | 8.23 | 4 |
| FY | 15.95 | 8.60 | 0.71 | 55.26 | 8.23 | 4 |
| FY | 15.45 | 7.75 | 0.51 | 55.26 | 8.23 | 4 |
| FY | 15.00 | 8.63 | 0.63 | 47.27 | 8.01 | 3 |
| FY | 14.68 | 8.48 | 0.55 | 47.27 | 8.01 | 3 |
| FY | 14.70 | 8.19 | 0.56 | 47.27 | 8.01 | 3 |
| FY | 15.10 | 9.08 | 0.68 | 52.88 | 7.96 | 4 |
| FY | 15.06 | 9.00 | 0.67 | 52.88 | 7.96 | 4 |
| FY | 15.87 | 8.96 | 0.71 | 52.88 | 7.96 | 4 |
| FY | 15.53 | 9.22 | 0.75 | 52.88 | 7.96 | 4 |
| FY | 15.50 | 8.87 | 0.67 | 52.66 | 8.21 | 5 |
| FY | 15.44 | 9.04 | 0.68 | 52.66 | 8.21 | 5 |
| FY | 14.20 | 8.64 | 0.60 | 52.66 | 8.21 | 5 |
| FY | 12.86 | 8.75 | 0.53 | 52.66 | 8.21 | 5 |
| FY | 11.54 | 8.03 | 0.39 | 52.66 | 8.21 | 5 |
| FY | 15.50 | 8.87 | 0.68 | 51.50 | 7.93 | 4 |
| FY | 16.11 | 9.10 | 0.73 | 51.50 | 7.93 | 4 |
| FY | 16.22 | 8.80 | 0.67 | 51.50 | 7.93 | 4 |
| FY | 13.74 | 8.23 | 0.50 | 51.50 | 7.93 | 4 |
| FY | 13.45 | 8.26 | 0.47 | 50.67 | 7.39 | 4 |
| FY | 13.68 | 8.34 | 0.54 | 50.67 | 7.39 | 4 |
| FY | 13.32 | 8.14 | 0.49 | 50.67 | 7.39 | 4 |
| FY | 13.36 | 8.12 | 0.53 | 50.67 | 7.39 | 4 |
| FY | 14.59 | 8.03 | 0.54 | 48.51 | 6.76 | 5 |
| FY | 12.26 | 7.36 | 0.43 | 48.51 | 6.76 | 5 |
| FY | 14.60 | 8.06 | 0.51 | 48.51 | 6.76 | 5 |
| FY | 13.17 | 7.79 | 0.47 | 48.51 | 6.76 | 5 |
| FY | 12.54 | 8.01 | 0.43 | 48.51 | 6.76 | 5 |
| FY | 11.85 | 7.61 | 0.39 | 51.30 | 8.14 | 4 |
| FY | 13.45 | 8.71 | 0.48 | 51.30 | 8.14 | 4 |
| FY | 13.06 | 7.92 | 0.47 | 51.30 | 8.14 | 4 |
| FY | 12.35 | 7.97 | 0.45 | 51.30 | 8.14 | 4 |
| FY | 14.53 | 8.11 | 0.57 | 48.84 | 7.00 | 4 |
| FY | 14.27 | 8.33 | 0.62 | 48.84 | 7.00 | 4 |
| FY | 14.94 | 8.19 | 0.60 | 48.84 | 7.00 | 4 |
| FY | 13.72 | 8.22 | 0.55 | 48.84 | 7.00 | 4 |
| FY | 15.34 | 8.65 | 0.65 | 51.51 | 7.92 | 6 |
| FY | 14.18 | 8.65 | 0.58 | 51.51 | 7.92 | 6 |
| FY | 15.18 | 8.35 | 0.62 | 51.51 | 7.92 | 6 |
| FY | 12.36 | 8.42 | 0.47 | 51.51 | 7.92 | 6 |
| FY | 12.48 | 8.70 | 0.50 | 51.51 | 7.92 | 6 |
| FY | 14.98 | 8.59 | 0.61 | 51.51 | 7.92 | 6 |
| FY | 14.08 | 8.60 | 0.58 | 49.55 | 7.77 | 4 |
| FY | 15.11 | 8.62 | 0.65 | 49.55 | 7.77 | 4 |
| FY | 13.84 | 8.88 | 0.54 | 49.55 | 7.77 | 4 |
| FY | 13.97 | 8.29 | 0.57 | 49.55 | 7.77 | 4 |
| FY | 14.50 | 8.92 | 0.61 | 48.60 | 6.81 | 4 |
| FY | 14.12 | 9.25 | 0.63 | 48.60 | 6.81 | 4 |
| FY | 15.17 | 8.08 | 0.59 | 48.60 | 6.81 | 4 |
| FY | 13.52 | 8.32 | 0.53 | 48.60 | 6.81 | 4 |
| FY | 13.72 | 8.36 | 0.55 | 53.36 | 8.62 | 4 |
| FY | 13.64 | 8.42 | 0.54 | 53.36 | 8.62 | 4 |
| FY | 13.67 | 8.35 | 0.53 | 53.36 | 8.62 | 4 |
| FY | 14.61 | 8.21 | 0.62 | 53.36 | 8.62 | 4 |
| FY | 18.28 | 9.84 | 0.76 | 49.00 | 8.05 | 3 |
| FY | 13.89 | 9.01 | 0.66 | 49.00 | 8.05 | 3 |
| FY | 14.97 | 9.14 | 0.60 | 49.00 | 8.05 | 3 |
| FY | 15.72 | 8.98 | 0.69 | 49.59 | 8.23 | 3 |
| FY | 16.50 | 8.68 | 0.68 | 49.59 | 8.23 | 3 |
| FY | 14.67 | 8.65 | 0.64 | 49.59 | 8.23 | 3 |
| FY | 16.77 | 8.75 | 0.70 | 50.61 | 7.54 | 4 |
| FY | 14.26 | 8.69 | 0.60 | 50.61 | 7.54 | 4 |
| FY | 14.68 | 8.66 | 0.63 | 50.61 | 7.54 | 4 |
| FY | 13.26 | 8.44 | 0.57 | 50.61 | 7.54 | 4 |
| FY | 15.82 | 8.87 | 0.80 | 47.77 | 7.78 | 3 |
| FY | 15.66 | 8.92 | 0.72 | 47.77 | 7.78 | 3 |
| FY | 17.13 | 8.61 | 0.72 | 47.77 | 7.78 | 3 |
| FY | 14.84 | 7.33 | 0.46 | 50.00 | 7.21 | 4 |
| FY | 12.93 | 7.97 | 0.47 | 50.00 | 7.21 | 4 |
| FY | 14.77 | 8.01 | 0.49 | 50.00 | 7.21 | 4 |
| FY | 15.10 | 7.76 | 0.54 | 50.00 | 7.21 | 4 |
| FY | 16.14 | 9.25 | 0.74 | 49.29 | 7.92 | 4 |
| FY | 17.59 | 9.66 | 0.77 | 49.29 | 7.92 | 4 |
| FY | 14.98 | 9.03 | 0.71 | 49.29 | 7.92 | 4 |
| FY | 16.34 | 9.51 | 0.72 | 49.29 | 7.92 | 4 |
| FY | 15.85 | 9.23 | 0.74 | 50.33 | 6.82 | 3 |
| FY | 14.54 | 9.03 | 0.76 | 50.33 | 6.82 | 3 |
| FY | 18.15 | 9.17 | 0.85 | 50.33 | 6.82 | 3 |
| FY | 19.48 | 8.94 | 0.90 | 51.92 | 8.04 | 2 |
| FY | 19.50 | 9.35 | 1.01 | 51.92 | 8.04 | 2 |
| FY | 14.16 | 8.80 | 0.49 | 48.40 | 7.59 | 3 |
| FY | 16.03 | 8.60 | 0.65 | 48.40 | 7.59 | 3 |
| FY | 17.57 | 8.66 | 0.73 | 48.40 | 7.59 | 3 |
| FY | 19.43 | 9.90 | 1.02 | 50.92 | 7.07 | 2 |
| FY | 17.25 | 9.14 | 0.77 | 50.92 | 7.07 | 2 |
| YN | 14.41 | 7.96 | 0.51 | 45.73 | 26.37 | 4 |
| YN | 14.07 | 8.00 | 0.54 | 45.73 | 26.37 | 4 |
| YN | 12.91 | 7.92 | 0.46 | 45.73 | 26.37 | 4 |
| YN | 13.33 | 8.12 | 0.52 | 45.73 | 26.37 | 4 |
| YN | 13.40 | 7.34 | 0.40 | 48.70 | 26.32 | 4 |
| YN | 14.28 | 6.94 | 0.41 | 48.70 | 26.32 | 4 |
| YN | 13.75 | 7.70 | 0.48 | 48.70 | 26.32 | 4 |
| YN | 13.65 | 7.95 | 0.50 | 48.70 | 26.32 | 4 |
| YN | 16.30 | 8.72 | 0.72 | 53.20 | 25.44 | 5 |
| YN | 14.18 | 8.56 | 0.57 | 53.20 | 25.44 | 5 |
| YN | 16.16 | 8.71 | 0.70 | 53.20 | 25.44 | 5 |
| YN | 14.93 | 9.20 | 0.65 | 53.20 | 25.44 | 5 |
| YN | 14.91 | 8.54 | 0.61 | 53.20 | 25.44 | 5 |
| YN | 13.52 | 7.99 | 0.53 | 48.58 | 24.31 | 4 |
| YN | 12.47 | 7.56 | 0.48 | 48.58 | 24.31 | 4 |
| YN | 13.78 | 8.08 | 0.49 | 48.58 | 24.31 | 4 |
| YN | 13.04 | 7.74 | 0.48 | 48.58 | 24.31 | 4 |
| YN | 14.50 | 8.25 | 0.62 | 51.52 | 24.65 | 3 |
| YN | 15.47 | 8.60 | 0.63 | 51.52 | 24.65 | 3 |
| YN | 15.98 | 8.44 | 0.63 | 51.52 | 24.65 | 3 |
| YN | 16.01 | 8.72 | 0.70 | 53.88 | 27.77 | 4 |
| YN | 15.52 | 8.71 | 0.66 | 53.88 | 27.77 | 4 |
| YN | 16.23 | 8.67 | 0.68 | 53.88 | 27.77 | 4 |
| YN | 15.97 | 8.67 | 0.69 | 53.88 | 27.77 | 4 |
| YN | 14.71 | 7.86 | 0.53 | 50.78 | 24.94 | 4 |
| YN | 14.59 | 8.24 | 0.51 | 50.78 | 24.94 | 4 |
| YN | 13.99 | 8.13 | 0.44 | 50.78 | 24.94 | 4 |
| YN | 14.08 | 8.10 | 0.49 | 50.78 | 24.94 | 4 |
| YN | 14.70 | 8.77 | 0.59 | 52.87 | 27.37 | 4 |
| YN | 14.48 | 8.73 | 0.57 | 52.87 | 27.37 | 4 |
| YN | 15.23 | 8.76 | 0.56 | 52.87 | 27.37 | 4 |
| YN | 16.13 | 8.90 | 0.66 | 52.87 | 27.37 | 4 |
| YN | 15.83 | 8.00 | 0.45 | 50.98 | 25.43 | 3 |
| YN | 14.45 | 8.22 | 0.44 | 50.98 | 25.43 | 3 |
| YN | 15.51 | 8.26 | 0.40 | 50.98 | 25.43 | 3 |
| YN | 12.26 | 6.39 | 0.33 | 52.71 | 27.62 | 4 |
| YN | 9.94 | 6.75 | 0.28 | 52.71 | 27.62 | 4 |
| YN | 15.04 | 8.58 | 0.38 | 52.71 | 27.62 | 4 |
| YN | 13.31 | 8.24 | 0.32 | 52.71 | 27.62 | 4 |
| YN | 15.82 | 7.50 | 0.62 | 44.14 | 23.01 | 3 |
| YN | 14.47 | 8.39 | 0.60 | 44.14 | 23.01 | 3 |
| YN | 15.74 | 8.09 | 0.71 | 44.14 | 23.01 | 3 |
| YN | 14.39 | 7.75 | 0.51 | 50.09 | 25.76 | 4 |
| YN | 14.72 | 8.23 | 0.52 | 50.09 | 25.76 | 4 |
| YN | 14.72 | 7.96 | 0.52 | 50.09 | 25.76 | 4 |
| YN | 14.62 | 8.32 | 0.53 | 50.09 | 25.76 | 4 |
| YN | 12.98 | 7.65 | 0.46 | 50.98 | 24.64 | 5 |
| YN | 14.64 | 8.25 | 0.59 | 50.98 | 24.64 | 5 |
| YN | 14.93 | 8.87 | 0.57 | 50.98 | 24.64 | 5 |
| YN | 15.02 | 8.31 | 0.56 | 50.98 | 24.64 | 5 |
| YN | 14.27 | 8.96 | 0.48 | 50.98 | 24.64 | 5 |
| YN | 12.31 | 7.77 | 0.44 | 54.44 | 28.61 | 4 |
| YN | 16.29 | 9.30 | 0.82 | 54.44 | 28.61 | 4 |
| YN | 15.24 | 8.62 | 0.69 | 54.44 | 28.61 | 4 |
| YN | 16.16 | 9.36 | 0.77 | 54.44 | 28.61 | 4 |
| YN | 14.64 | 8.25 | 0.59 | 55.40 | 30.68 | 4 |
| YN | 14.93 | 8.87 | 0.57 | 55.40 | 30.68 | 4 |
| YN | 15.02 | 8.31 | 0.56 | 55.40 | 30.68 | 4 |
| YN | 14.27 | 8.96 | 0.48 | 55.40 | 30.68 | 4 |
| YN | 17.04 | 8.68 | 0.65 | 53.46 | 26.35 | 4 |
| YN | 16.99 | 8.84 | 0.70 | 53.46 | 26.35 | 4 |
| YN | 17.35 | 8.57 | 0.70 | 53.46 | 26.35 | 4 |
| YN | 16.22 | 8.50 | 0.60 | 53.46 | 26.35 | 4 |

Notes: BT: Beitun population; FY: Fuyun population; YN: Yining population; SVL: Snout-vent length; CS: clutch mass; TBW: Tail base width; EL: Egg length; EW: Egg width; EM: Egg mass.
