# Supplementary material for: Variations in the reproductive strategies of three populations of Phrynocephalus helioscopus in China
Source: PeerJ. 2018 Oct 24;6:e5705. doi: 10.7717/peerj.5705 (PMC6203940; doi:10.7717/peerj.5705)
Supplement: Table S2 [file peerj-06-5705-s002.docx]

| Population | SVL | BM | CS | CM | RCM |
| --- | --- | --- | --- | --- | --- |
| BT | 42.83 | 5.33 | 2 | 1.1 | 0.21 |
| BT | 43.94 | 3.21 | 3 | 1.43 | 0.45 |
| BT | 45.24 | 2.71 | 3 | 1.75 | 0.65 |
| BT | 45.51 | 3.19 | 3 | 1.56 | 0.49 |
| BT | 45.51 | 2.63 | 4 | 1.97 | 0.75 |
| BT | 46.15 | 2.34 | 2 | 1.19 | 0.51 |
| BT | 46.34 | 2.68 | 2 | 1.05 | 0.39 |
| BT | 46.7 | 3.97 | 2 | 1.14 | 0.29 |
| BT | 46.72 | 3.07 | 3 | 1.54 | 0.5 |
| BT | 46.8 | 4.1 | 2 | 0.93 | 0.23 |
| BT | 46.99 | 4.18 | 3 | 1.48 | 0.35 |
| BT | 47.75 | 4.28 | 2 | 1.33 | 0.31 |
| BT | 48 | 4.13 | 4 | 1.3 | 0.31 |
| FY | 47.27 | 4.32 | 3 | 1.74 | 0.4 |
| FY | 47.77 | 3.85 | 3 | 2.24 | 0.58 |
| FY | 48.4 | 3.65 | 3 | 1.87 | 0.51 |
| FY | 48.42 | 5.5 | 4 | 2.03 | 0.37 |
| FY | 48.51 | 3.09 | 5 | 2.38 | 0.77 |
| FY | 48.6 | 3.98 | 4 | 2.36 | 0.59 |
| FY | 48.84 | 3.78 | 4 | 2.34 | 0.62 |
| FY | 49 | 4.33 | 3 | 2.02 | 0.47 |
| FY | 49.29 | 4.68 | 4 | 2.94 | 0.63 |
| FY | 49.55 | 4.81 | 4 | 2.34 | 0.49 |
| FY | 49.59 | 5.01 | 3 | 2.01 | 0.4 |
| FY | 50 | 3.6 | 4 | 1.96 | 0.54 |
| FY | 50.33 | 3.94 | 3 | 2.35 | 0.6 |
| FY | 50.61 | 4.45 | 4 | 2.5 | 0.56 |
| FY | 50.67 | 3.93 | 4 | 2.03 | 0.52 |
| FY | 50.92 | 4.78 | 2 | 1.79 | 0.37 |
| FY | 51.3 | 3.76 | 4 | 1.79 | 0.48 |
| FY | 51.5 | 4.7 | 4 | 2.58 | 0.55 |
| FY | 51.51 | 6.03 | 6 | 3.43 | 0.57 |
| FY | 51.92 | 5.84 | 2 | 1.91 | 0.33 |
| FY | 52.66 | 5.49 | 5 | 2.87 | 0.52 |
| FY | 52.88 | 6.39 | 4 | 2.81 | 0.44 |
| FY | 53.36 | 4.38 | 4 | 2.24 | 0.51 |
| FY | 55.26 | 4.9 | 4 | 2.61 | 0.53 |
| YN | 44.14 | 4.78 | 3 | 1.93 | 0.4 |
| YN | 45.73 | 4.02 | 4 | 2.03 | 0.5 |
| YN | 48.58 | 4.01 | 4 | 1.98 | 0.49 |
| YN | 48.7 | 3.23 | 4 | 1.79 | 0.55 |
| YN | 50.09 | 5.03 | 4 | 2.08 | 0.41 |
| YN | 50.78 | 4.07 | 4 | 1.97 | 0.48 |
| YN | 50.98 | 3.73 | 3 | 1.29 | 0.35 |
| YN | 50.98 | 5.96 | 5 | 2.66 | 0.45 |
| YN | 51.52 | 4.24 | 3 | 1.88 | 0.44 |
| YN | 52.71 | 5.2 | 4 | 1.31 | 0.25 |
| YN | 52.87 | 5.85 | 4 | 2.38 | 0.41 |
| YN | 53.2 | 4.98 | 5 | 3.25 | 0.65 |
| YN | 53.46 | 4.63 | 4 | 2.65 | 0.57 |
| YN | 53.88 | 5.46 | 4 | 2.73 | 0.5 |
| YN | 54.44 | 5.43 | 4 | 2.72 | 0.5 |
| YN | 55.4 | 5.96 | 4 | 2.2 | 0.37 |

Notes: BT: Beitun population; FY: Fuyun population; YN: Yining population; SVL: Snout-vent length; CS: clutch mass; CM: Clutch mass; RCM: Relative clutch mass; BM: female post-oviposition mass
